# Epigenetic biomarker screening by FLIM-FRET for combination therapy in ER+ breast cancer

**DOI:** 10.1186/s13148-019-0620-6

**Published:** 2019-01-30

**Authors:** Wenjie Liu, Yi Cui, Wen Ren, Joseph Irudayaraj

**Affiliations:** 1 Department of Agricultural and Biological Engineering, Purdue University, West Lafayette, IN 47907 USA; 2 Bindley Bioscience Center, Purdue University, West Lafayette, 47907 IN USA; 3 Present Address: Department of Bioengineering, Cancer Center at Illinois, Micro and Nanotechnology Laboratory, University of Illinois at Urbana-Champaign, Champaign, IL 61801 USA; 4 Earth and Biological Science Directorate, Pacific Northwest National Laboratory, Richland, WA 99354 USA

**Keywords:** Fluorescence lifetime, Förster resonance energy transfer, Epigenetic markers, Combination therapy, Breast cancer, Histone acetylation, Anacardic acid

## Abstract

Hormone-dependent gene expression involves dynamic and orchestrated regulation of epigenome leading to a cancerous state. Estrogen receptor (ER)-positive breast cancer rely on chromatin remodeling and association with epigenetic factors in inducing ER-dependent oncogenesis and thus cell over-proliferation. The mechanistic differences between epigenetic regulation and hormone signaling provide an avenue for combination therapy of ER-positive breast cancer. We hypothesized that epigenetic biomarkers within single nucleosome proximity of ER-dependent genes could serve as potential therapeutic targets. We described here a Fluorescence lifetime imaging-based Förster resonance energy transfer (FLIM-FRET) methodology for biomarker screening that could facilitate combination therapy based on our study. We screened 11 epigenetic-related markers which include oxidative forms of DNA methylation, histone modifications, and methyl-binding domain proteins. Among them, we identified H4K12acetylation (H4K12ac) and H3K27 acetylation (H3K27ac) as potential epigenetic therapeutic targets. When histone acetyltransferase inhibitor targeting H4K12ac and H3K27ac was combined with tamoxifen, an enhanced therapeutic outcome was observed against ER-positive breast cancer both in vitro and in vivo. Together, we demonstrate a single molecule approach as an effective screening tool for devising targeted epigenetic therapy.

**Electronic supplementary material:**

The online version of this article (10.1186/s13148-019-0620-6) contains supplementary material, which is available to authorized users.

## Introduction

Breast cancer poses a tremendous burden on health care due to its high prevalence and mortality [1, 2]. Even though substantial progress has been made to detect the disease early and prolong patient survival, currently available treatments are far from satisfactory [3, 4]. Of the breast cancer population, around 80% of patients bear ER-positive tumors, and this makes hormone therapy that block the estrogen pathways a standard option of treatment [5]. However, long-term hormonal therapy would result in side effects and even tumor recurrence which are resistant to subsequent treatments. Such inadequacies require more in-depth evaluation and optimization of therapies [6, 7]. Targeted molecular therapy provides an alternative to further improve the outcomes of conventional treatments such as surgery and chemotherapy [8]. To screen for molecular targets in cancer therapy, traditional methods rely on established libraries comprising of tens of thousands of compounds to assess their effects on a model cell line, which are too empirical, low in efficiency, and do not account for molecular interaction [9, 10]. Direct interactions between ER and various epigenetic modifications could provide insights into its role in breast cancer development as a potential therapeutic target. Such interactions could be readily and precisely evaluated by fluorescence lifetime imaging-based Förster resonance energy transfer (FLIM-FRET) at single molecule resolution.

FLIM-FRET allows for the observation of interaction and other proximity-based evaluation directly unlike intensity-based FRET which is susceptible to reversible photobleaching or photoconversion of donor molecules [11]. In drug discovery, FRET screening has been used to identify molecules or drugs that dissociate DNA-protein or protein-protein interactions in search of possible inhibitors [12, 13]. Other applications of FRET include evaluation of apoptosis or protein aggregation for drug screening with known anticancer molecules [14, 15]. These applications focus on FRET screening of known compounds and have not been utilized to discover novel therapeutic targets.

Herein, we evaluate the interaction of epigenetic targets with ERα by FLIM-FRET to reveal secondary therapeutic targets. Based on our screening results, we find that H3K27 acetylation (H3K27ac) and H4K12 acetylation (H4K12ac) usually co-localize with ERα at the nanoscale resolution and could serve as potential therapeutic targets. Therefore, we assessed the effects of histone acetyltransferase inhibitor (HATi) on these potential epigenetic targets. Our results show that a combination of tamoxifen and anacardic acid (AA) resulted in significant tumor suppression on MCF7 cell growth both in vitro and in mice xenograft than treatment with either drug alone. Anacardic acid not only reduces the amount of these epigenetic modifications in vitro but also inhibits H4K12ac-estrogen receptor element (ERE) interactions, revealing a possible mechanism of action of AA in breast cancer treatment. As proof of principle, we demonstrated that a FLIM-FRET platform could serve as a robust and reliable method for discovering novel epigenetic therapeutic targets in ER-dependent breast cancer. Moreover, the combination of tamoxifen and anacardic acid could potentially yield a promising therapeutic strategy for ER-positive breast cancers.

## Results and discussion

### FLIM-FRET screening identifies epigenetic biomarker candidates for therapy

**Fig. 1:**
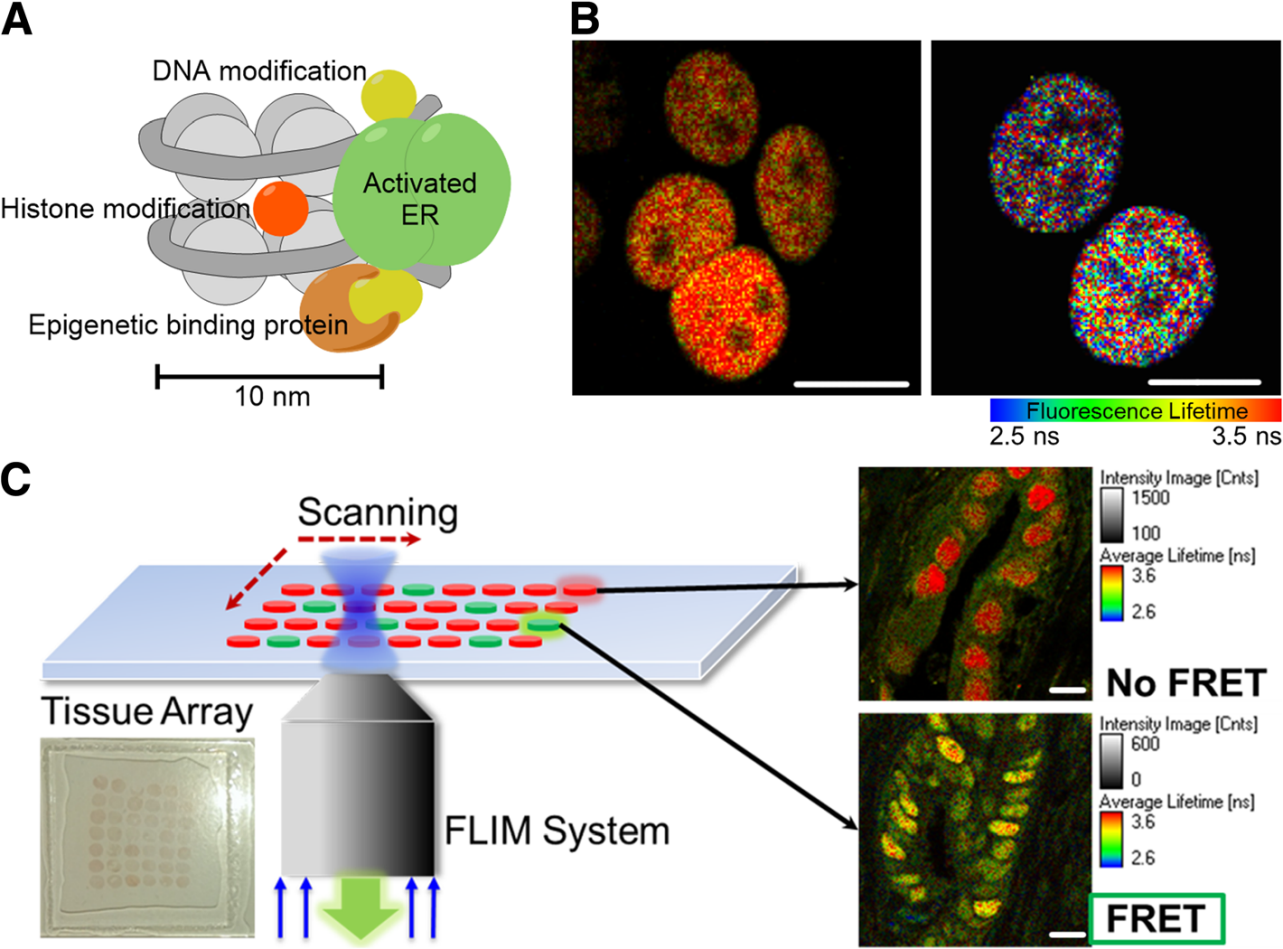
FLIM-FRET screening identifies potential epigenetic treatment targets. **a** Schematic illustration of possible ERα-epigenetic biomarker association in a nucleosome scale that facilitates ER-regulated gene transcription. **b** Typical FLIM images show control (left) and FRET (right) results as lifetime reduces with distance between donor and acceptor fluorophores. **c** Schematic and prototype of biopsy tissue arrays subjected to FLIM analysis

**Fig. 2:**
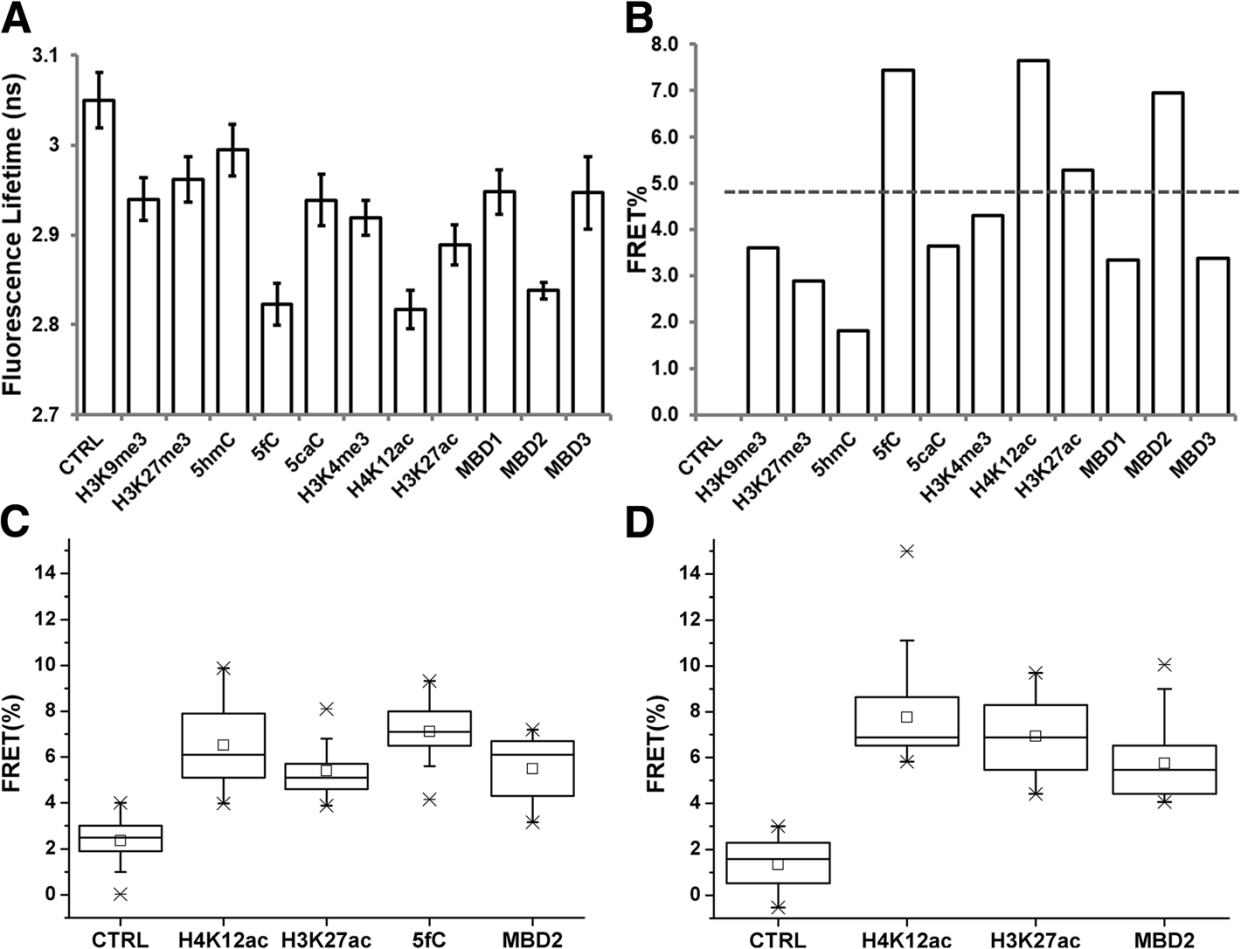
FLIM-FRET screening results from MCF7 cell and patient tissue. **a** Summarized lifetime information of the FRET donor (mean ± s.d.). **b** Calculated average FRET efficiency based on lifetime values. FRET efficiency of 5% is set as the threshold. **c** FLIM-FRET screening results from MCF7 cell immunostaining indicates H4K12ac, H3K27ac, MBD2, and 5fC are in close proximity with ERα. Data from 3 independent immunostains with 15 cells analyzed in each experiment. **d** FLIM-FRET screening from ER+ patient tissue array shows H4K12ac, H3K27ac, and MBD2 as potential treatment targets, *n* = 30. Data shown as boxplot with values of minimum, 5% percentile, 25% percentile, median, 75% percentile, maximum, and mean (center square marks)

We first assessed the local epigenetic changes associated with ERα in MCF7 cells and ER-positive tumor tissue samples from patients. The co-localization of ERα and epigenetic biomarkers evaluated by FRET suggests a possible interaction and plays a role in ER binding to ERE genes to induce cancer cell proliferation (Fig. 1a). Utilizing FLIM-FRET, we evaluated the molecular interactions of 11 different epigenetic relevant targets for possible association with ERα in MCF7 cells. We set the FRET efficiency threshold to above 5% for target identification, which indicates that the targets (ERα and epigenetic marks) are co-localized within 10 nm from each other. A typical FRET-induced fluorescence lifetime change in the ERα–ALEXA488 complex is shown in Fig. 1b. FRET analysis of the 11 epigenetic markers with ERα based on immunostaining was performed to determine possible molecular interactions as epigenetic drug targets (Fig. 2a, b, Additional file 1: Figure S1A). Results from quantitative screening suggest that 5-formylcytosine (5-fC), methyl-CpG-binding domain protein 2 (MBD2), H3K27ac, and H4K12ac are potential candidates for epigenetic therapy (Fig. 2c).

To further facilitate the screening outcome, we evaluated patient tissue arrays for H4K12ac, H3K27ac, and MBD2 (Fig. 1c, Additional file 1: Figure S1B). We observed a consistent outcome for all of these targets to be within 10 nm from ERα (Fig. 2d). The tissue-screening array showed excellent potential for high throughput screening and automation with further investigation. It has been reported that MBD2 is required to activate pro-metastatic genes in breast cancer and to meditate the repression of methylated tumor suppressor genes, indicating a putative role of MBD2 in breast cancer [16, 17]. However, to the best of our knowledge, no commercially available small molecule inhibitor targets MBD2 or 5fC and hence targeting these markers is beyond the scope of this work. On the other hand, histone acetyltransferase inhibitors (HATi) can readily downregulate specific histone acetylation levels [18]. Our results suggest that H4K12ac and H3K27ac are potential candidates for epigenetic treatment in ER-positive breast cancer.

The co-existence of ERα-epigenetic marks suggests a potential role of these epigenetic targets in ER-dependent gene transcription. Indeed, an elevated level of H4K12ac was observed with E2 treatment [19, 20]. Moreover, recent studies showed that H4K12ac had increasing occupancy near the transcription start sites of estrogen-induced genes and had a stronger correlation with the ER-positive phenotypes [21]. E2 stimulated H3K27ac was abundant at the distal ERE of TFF1 gene, suggesting a potential recruiting role of H3K27ac at ERE [22–24]. These findings support our FLIM-FRET screening results which implicate H4K12ac and H3K27ac as potential targets in ER+ breast cancer. Our data showed that FLIM-FRET screening provides a fast and sensitive tool for discovering novel epigenetic therapeutic targets in ER+ breast cancer.

### Combination therapy based on FLIM-FRET screening

**Fig. 3:**
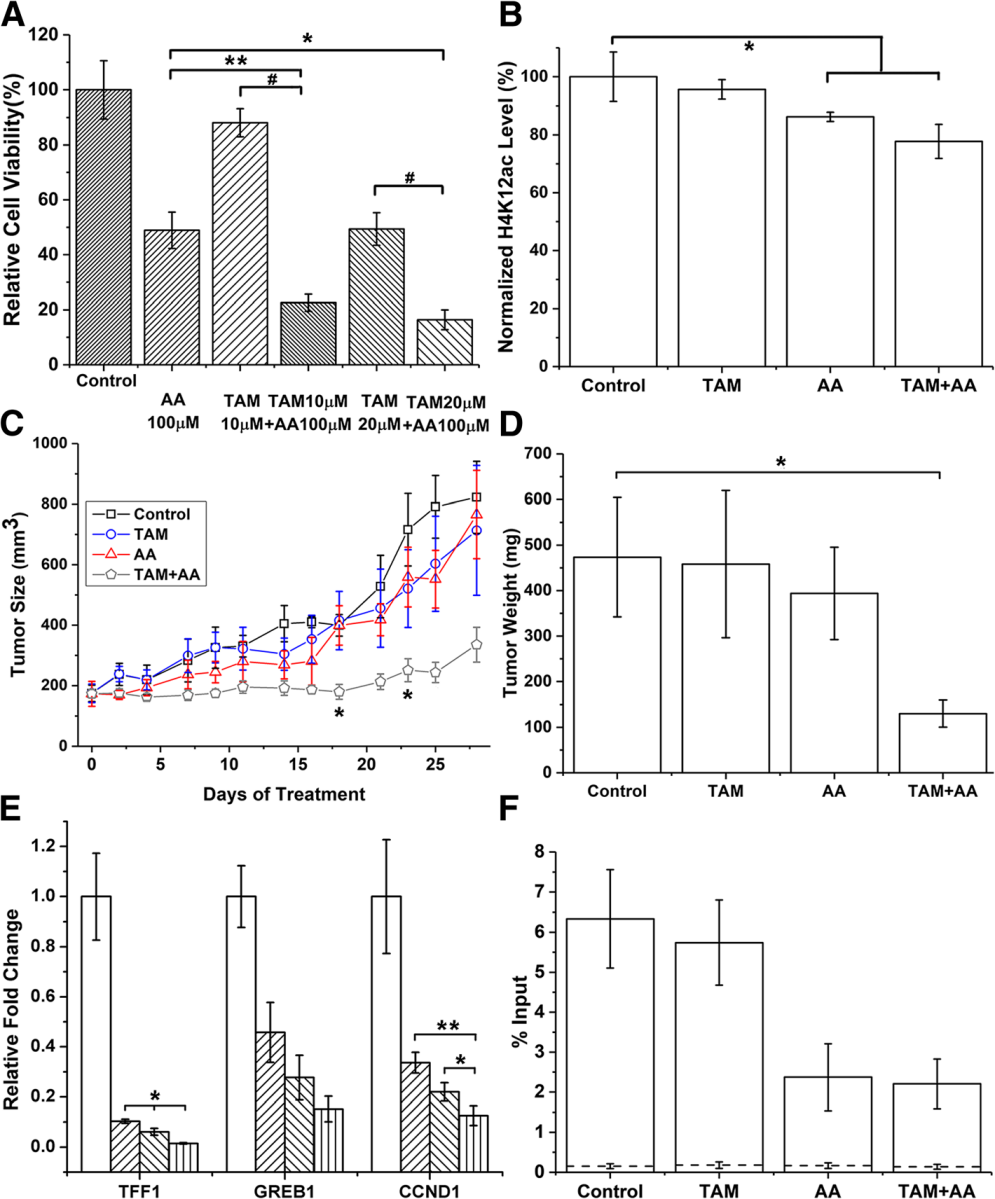
Combination treatment based on FLIM-FRET screening. **a** Co-treatment of anacardic acid and tamoxifen affects MCF7 cell viability. After 48 h of treatment, co-treatment shows enhanced inhibition on cell viability. Each drug treatment was done in quadruplicate and values are averaged (*n* = 5, mean ± s.d.). **b** Global histone acetylation quantification of H4K12ac. MCF7 cells were treated with 10 μM tamoxifen and 50 μM anacardic acid for 24 h. Histone protein were extracted and assayed. Values are shown as mean ± s.d. from three independent experiments. **c** Tumor volume of MCF7 mice xenograft with co-treatment of tamoxifen (4 mg kg^−1^) and anacardic acid (1 mg kg^−1^) for a month. * indicates that the co-treatment group is significantly different from all other groups. (*n* = 5, mean ± s.e.m). **d** Tumor weight measured at the end of 1-month treatment. (*n* = 5, mean ± s.e.m). **e** MCF7 cells treated with tamoxifen and anacardic acid; the transcription level of three ER responsive genes was determined by qRT-PCR (*n* = 4, mean ± s.d.). Group from left to right as control , TAM 10 μM , AA 100 μM , and co-treatment of TAM and AA . **f** qRT-PCR was done with mice tumor for H4K12ac occupancy near GREB1 ERE in CHIP samples. CHIP-negative control with IgG antibody is shown as dashed line. (*n* = 2), values are mean ± s.d. of two independent experiments. *p < 0.05, **p < 0.01, #*p* < 0.0001

Studies that implicate the association of histone acetylation and methylation in breast cancer development have grown over the years [25–27]. However, the potential therapeutic targets were less clearly defined and understood. Thus, we assessed whether a combination of tamoxifen (TAM) [28] and epigenetic intervention against our screened targets with histone acetyltransferase inhibitor (HATi) could provide a better therapeutic outcome as revealed by cell proliferation assays and mice xenograft studies. Dose-dependent effect of anacardic acid is shown in Additional file 2: Figure S2A. Our experiments revealed that anacardic acid inhibits MCF7 cell viability at a higher concentration than reported [29], possibly due to slight difference in cell culture and source of anacardic acid. When tamoxifen at either 10 or 20 μM is combined with 100 μM anacardic acid, MCF7 cell viability was significantly inhibited in both cases to 22.5 and 16.4%, respectively (Fig. 3a). To further support our findings, experiments utilizing T47D cells also indicated a similarly enhanced therapeutic effect (Additional file 2: Figure S2B). Anacardic acid significantly reduced the level of H3K27ac and H4K12ac in vitro and in vivo (Fig. 3b, Additional file 2: Figure S2C-F). Loss of FRET efficiency between ERα and histone acetylation was observed with anacardic acid treatment (Additional file 3: Figure S3) possibility because loss of the association between ERα and histone acetylation. On the other hand, non-specific regulation by other HATi did not show effective inhibition of cell proliferation (Additional file 4: Figure S4). Thus, we show that FLIM-FRET screening is accurate and robust in discovering novel epigenetic targets at the nucleosome level.

To validate and assess the clinical relevance, we further tested the inhibitory effect of combination treatment in NSG mice bearing MCF7 cells. Anacardic acid combined with tamoxifen showed a significant reduction in tumor growth compared to the control groups after 18 days of treatment, and the measured tumor size was maintained at near baseline tumor volumes (Fig. 3c, d), whereas shorter exposure or reduced concentration exhibited a lower therapeutic effect (Additional file 5: Figure S5A-C). No significant difference in tumor weight between the different groups was observed suggesting a combination treatment without noticeable toxicity (data not shown). Tamoxifen is known to have cytotoxicity effect in normal cells [30, 31], whereas anacardic acid has been shown to have little effect on cell proliferation [29, 32], suggesting that a combination treatment will likely reduce the side effect from either drug alone.

The next step was to test whether the combinatorial effect was due to the enhanced inhibition of ER-regulated gene transcription. qRT-PCR was thus applied to investigate the effect of tamoxifen and anacardic acid on the transcription of established ER-induced genes: CCND1, TFF1, and GREB1 (Fig. 3e, Additional file 5: Figure S5D). We observed enhanced inhibition from combination treatment than either treatment alone. To directly assess whether anacardic acid regulates gene expression by altering histone acetylation, qRT-PCR was performed on the chromatin immunoprecipitated samples to examine H4K12ac occupancy near GREB1 promoter region. We observed that anacardic acid with or without tamoxifen reduced H4K12ac occupancy near the GREB1 transcription starting site whereas tamoxifen alone did not exhibit this effect (Fig. 3f). The co-presence of ERα and H4K12ac at both GREB1 and TFF1 promoter region further validates our FLIM-FRET screening results (Additional file 6: Figure S6).

Others have proposed a possible mechanism wherein anacardic acid inhibits ER–ERE binding, but have also observed that anacardic acid inhibits ER-negative tumor growth [29], suggesting the involvement of other mechanisms, such as nuclear factor-kappa B signaling pathway [33]. Here, we show a possible mechanism of action of the effect of anacardic acid by inhibiting p300 HAT activity, which in turn reduces the level of H4K12/H3K27ac and decreases the occupancy near ERE gene transcription start site. However, we cannot rule out the involvement of other histone acetylation markers, which may also be affected by anacardic acid; hence, a broader screening is warranted in future studies.

Together, our results suggest that a combination treatment of anacardic acid and tamoxifen can regulate ER-responsive gene via different mechanisms. Tamoxifen binds to ER and thus inhibits ER-DNA activity [7] whereas anacardic acid disrupts H4K12ac-ERE association. Our studies suggest that a FLIM-FRET screening approach can be used to discover new epigenetic targets which we demonstrate with ER+ breast cancers as a test study. The FLIM-FRET platform provides a fast and highly sensitive approach to identify ER-related epigenetic targets to guide new combination therapy based on the identified epigenetic biomarkers successfully. Since FLIM-FRET can yield single molecule sensitivity [34, 35], the approach proposed is highly sensitive compared to any of the traditional screening approaches to date [12, 15]. In the future, with an automated multi-well FLIM-FRET device, numerous epigenetic targets can be screened simultaneously to achieve high throughput with significantly increased screening efficiency. The proposed approach can also be applied to screen a range of other nuclear receptor-related targets not only for cancer but also for other diseases as well.

## Materials and methods

### Cell culture

MCF7 cells (ATCC) were regularly cultured in Dulbecco’s modified Eagle’s medium (DMEM) supplemented with 10% fetal bovine serum (Atlanta Biologicals), 100 IU/ml penicillin and 100 μg/ml streptomycin in a humidified incubator with 5% CO_2_ at 37 °C. Cells were sub-cultured in phenol red-free DMEM with 10% dextran-coated charcoal-stripped fetal bovine serum (CS-FBS) (Atlanta Biologicals) and within 2 to 5 passages.

T47D cells were cultured in Rosewell Park Memorial Institute medium (RPMI) supplemented with 10% fetal bovine serum, 100 IU/ml penicillin, and 100 μg/ml streptomycin. Cells were sub-cultured in phenol red-free RPMI with 10% CS-FBS for experiments and within 10 passages.

### Immunofluorescence staining

Cells were fixed with 4% paraformaldehyde for 15 min and rinsed with 1× phosphate-buffered saline (PBS) buffer. Cell membrane was permeabilized by 0.5% Triton X-100 (Sigma) for 20 min and rinsed with PBS for 10 min. After DNA denaturation treatment with HCl (optional step for staining 5hmC/5fC/5caC), cells were then blocked with PBS containing 5% goat serum and 0.3% Triton X-100 for 1 h. Primary antibodies were diluted in PBS containing 1% BSA and 0.3% Triton X-100 then added to cells and incubated overnight at 4 °C. The following day cells were rinsed with PBS. Then, samples were incubated with the corresponding diluted secondary antibodies for 1 h and washed with PBS before FLIM imaging. Primary antibodies used in this study are histone H3K9me3 (39765, Active Motif), histone H3K27me3 (39155, Active Motif), 5-hydroxymethylcytosine (5-hmC) (39791, Active Motif), 5-Formylcytosine (5-fC) (61227, Active Motif), 5-Carboxylcytosine (5-caC) (61229, Active Motif), anti-histone H3 tri-methyl K4 (ab8580, Abcam), anti-histone H4 acetyl K12 (ab46983, Abcam), histone H3K27ac (pAb39133, Active Motif), anti-MBD1 (ab108510, Abcam), anti-MBD2 (ab38646, Abcam), anti-MBD3 (ab115692, Abcam), and ERα (sc-8002, Santa Cruz). Antibodies were diluted as suggested by vendor recommendation. Secondary antibodies are goat anti-mouse Alexa Fluor 546 (1:2000) and goat anti-rabbit Alexa Fluor 488 (1:2000) (Invitrogen, A11071, and A11070). For tissue immunostaining, cryo-sectioned breast cancer tissue (ERα+) slices (5 μm thick) were fixed with fresh formalin and embedded in paraffin (provided by Dr. Sunil Badev at Indiana University School of Medicine under the existing protocol). Then, tissue slides were deparaffinized with xylene solutions and rehydrated with ethanol series. Heat-induced epitope retrieval was carried out in boiling Tris-EDTA buffer (TBS, pH = 9.0) for 20 min, followed by thorough washing with 0.025% Triton X-100. DNA was denatured with 1 M HCl for 30 min at 37 °C (staining 5hmC/5fC/5caC). The tissue slides were subsequently blocked with TBS buffer containing 1% BSA and 10% goat serum. Then, the slides were incubated with 5 nM primary antibodies in a humid chamber overnight at 4 °C. The next day, 1:2000 diluted secondary antibodies were applied for 1 h at room temperature. After washing with fresh PBS buffer, tissue slides were sealed in VECTASHIELD mounting medium (Life Technologies) for FLIM imaging.

### Fluorescence lifetime imaging-based Förster resonance energy transfer

Confocal fluorescence lifetime imaging (FLIM) was performed with a Microtime200 (Picoquant GmbH) or Alba (ISS, Champaign) scanning confocal time-resolved microscope system. Details of the instrumentation are as described previously [35]. For Microtime200, a 465 nm picosecond pulsed laser at 40 MHz repetition rate was used to excite ALEXA488 antibody through an apochromatic water immersion objective (× 60, NA = 1.2). Photons were collected by the same objective and passed through a 50 μm pinhole and a band-pass filter (520/40 nm, Chroma) before reaching an avalanche photodiode (SPCM-AQR, PerkinElmer Inc.). Detected photons were stored in time-tagged time-resolved (TTTR) format to generate time-correlated single-photon counting (TCSPC) histograms (TimeHarp 200, Picoquant). The laser power was controlled to have a peak photon value of ~ 10^4^ in the TCSPC histogram. Fluorescence lifetime (*τ*) was obtained by fitting the TCSPC decay pattern as *F*(*t*) = *F*_0_*e*^−*t*/*τ*^,(1).

For Alba, a 488 nm picosecond pulsed laser at 20 MHz repetition rate was used to excite ALEXA 488 antibody through an apochromatic water immersion objective (× 60, NA = 1.2). Photons were collected by the same objective, reflected by a 560 dichroic filter (Chroma) and passed through a 50 μm pinhole and a band-pass filter (525/50, Chroma) before reaching an avalanche photodiode. Detected photons were stored in TTTR format and generate a TCSPC histogram (Becker and Hickl).

Förster resonance energy transfer (FRET) was used to probe potential molecular interaction between epigenetic modifications and ERα by evaluating the distance between them. ERα-ALEXA 488 and various epigenetic modification targeting antibody-ALEXA 546 complexes were prepared, and the FRET efficiency was calculated based on the following equation: $$ E=1-\raisebox{1ex}{${\tau}_{da}$}\!\left/ \!\raisebox{-1ex}{${\tau}_d$}\right. $$, (2) where *τ*_*da*_ is the lifetime of dual antibodies labeled cells, and *τ*_*d*_ is the lifetime of the donor only cells [36–38].

### MTT cell viability assays

Approximately, 7000 MCF7 cells were plated per well in the 96 well supplied with phenol-red-free DMEM, 10% CS-FBS, 100 IU/ml penicillin and 100 μg/ml streptomycin. Similarly, T47D cells were cultured with phenol-red-free RPMI, 10% CS-FBS, and antibiotics. After 24 h of incubation, various concentration of tamoxifen, CPTH2, MB-3, and anacardic acid were added and incubated for 48 h. All drugs were purchased from Sigma-Aldrich, dissolved in DMSO and aliquoted into small tubes and stored at − 20 °C. At the end of the treatments, 20 μl of 3-(4,5-dimethylthiazol-2-yl)-2,5-diphenyltetrazolium bromide (MTT) was added to each well from a 5 mg/ml stock in PBS, and the 96-well plate was incubated for 3.5 h. Cell culture medium is removed carefully, and 150 μl of MTT solvent composed of 4 mM HCl, 0.1 (*v*/*v*) % IGEPAL CA-630 in isopropanol was added to each well. The plate was placed on a shaker for 15 min before reading the absorbance at 570 nm. Wells without any cells but with MTT solvent was used as the background. Background absorbance was averaged, and then subtracted for all other readings. Within each experiment, drug treatments were done in quadruplicate, and the values were averaged.

### Global histone acetylation quantification

Drug-treated cells and mice xenograft tissue were trypsinized and harvested for histone extraction with EpiQuik Total Histone Extraction Kit (Epigentek). Extracted histone protein was assayed on 18% SDS-PAGE gel and stained by Coomassie Blue. Total histone protein concentrations were measured with BCA Protein Assay Kit (Pierce) in triplicate. Quantitative detection of H4K12ac or H3K27ac changes were performed with EpiQuik Global Acetyl-Histone H4-K12/H3K27 Quantification Kit (Epigentek) according to the manufacturer’s instructions.

Histone protein extraction was electrophoresed on 12% Mini-PROTEAN protein gels (Bio-rad) and transferred to polyvinylidene difluoride (PVDF) membrane (Bio-rad). After incubation with 5% non-fat milk for 1 h, the blots were probed with H4K12ac antibody (1:1000, Santa Cruz Biotechnology) or H4 antibody (1:1000, Santa Cruz Biotechnology) in Tris-Buffered Saline with Tween 20 (TBST) plus 5% non-fat milk at 4 °C overnight. HRP conjugated goat anti-mouse antibody was used as the secondary antibody.

### Quantitative RT-PCR

Cells ~ 10,000 cell/ml were plated in T25 flasks supplied with DMEM (no phenol red), 10% CS-FBS, 100 IU/ml penicillin, and 100 μg/ml streptomycin for 2 days. Then, drugs at various concentrations were added, and cells were incubated for 48 h before trypsinization. RNA extraction from cells or tumor tissues were performed with RNeasy Mini Kit (Qiagen) followed by reverse transcription with iScriptTM cDNA Synthesis Kit (Bio-Rad) per manufacturer’s instructions. Polymerase chain reaction (PCR) amplification was performed in a StepOnePlusTM system (Applied Biosystems) with SYBR® Green PCR Master Mix (Life Technologies). The ΔΔCt method was used to determine the fold change of gene transcription level and normalized against the control gene GAPDH. GAPDH-F: CAGCCTCAAGATCATCAGCA; GAPDH-R: TGTGGTCATGAG TCCTTCCA; GREB1-F: CAAAGAATAACCTGTTGGCCCTGC; GREB1-R: GACATGCCTGCGCTCTCATACTTA; CCND1-F: TGGAGGTCTGCGAGGAACAGAA; CCND1-R: TGGAGGTCTGCGAGGAACAGAA; TFF1-F: GAACAAGGTGATCTGCG and TFF1-R: TGGTATTAGGATAGAAGCACCA were designed based on Primer-BLAST online.

### Mice xenograft

All animal experiments were performed under an approved Purdue Animal Care and Use Committee (PACUC) protocol in an AAALAC-accredited facility with 24/7 veterinary care. Eleven to fifteen-week-female NSG mice (19–25 g) were acquired from Jackson Labs BESR colony. Mice were kept under standardized conditions with 50 ± 10% relative humidity, 20 ± 1 °C, and a 12 h light/dark cycle. Sixty-day-releasing 17β-Estradiol (E2) pellets (0.36 mg/pellet, Innovative Research of America) were inserted on the shoulders subcutaneously via a trocar. Around 3 × 10^6^ MCF7 cells (ATCC) were subcutaneously inoculated into the right flank of the animals, respectively. After the tumor length reached ~ 7 mm, these mice were randomly divided into six groups and treatment was initiated. Drugs were dissolved in PBS in small aliquots and frozen at − 20 °C in the dark before usage. Mice were IP injected with drugs at the dosage based on mice weight (control group 100 μl of 3% DMSO in PBS, Tamoxifen: 4 mg kg^−1^, anacardic acid 0.3 mg kg^−1^ or 1 mg kg^−1^). Tumor xenograft volumes were measured using calipers and calculated using the equation *L* × *W*^2^/2, where *W* is tumor width, and *L* is tumor length.

### Chromatin immunoprecipitation

Chromatin immunoprecipitation (ChIP) assays were performed using EpiQuik Tissue Chromatin Immunoprecipitation Kit (Epigentek) according to the instructions supplied with minor modification per manufacturer’s suggestions. Around 50 mg of tissue was cut into 1–2 mm^3^ pieces and cross-linked with 1% formaldehyde for 15 min at room temperature on a rocking platform. Subsequent cell lysis and DNA shearing were completed before incubation of tissue extracts with ERα (F-10X, Santa Cruz), anti-histone H4 acetyl K12 (ab46983, Abcam) or normal mouse IgG (Epigentek). After protein/DNA immunoprecipitation, reversal of cross-linked DNA was performed, and DNA was purified using a kit-supplied reagent (Epigentek). Primer for GREB1 [21] qRT-PCR: Forward-5′-GCCAAATGGAAGAAGGACAG-3′; Reverse-5′-ACCACCTACCTCCAGTCACC-3′ were used. Primer for TFF1 [39, 40]: Forward-5′-GGCAGGCTCTGTTTGCTTAAAGAGCG-3′; Reverse-5′-GGCCATCTCTCACTATGAATCACTTCTGC-3′.

### Statistical analysis

Two-tailed Student’s *t* tests were performed to determine the difference between averaged mean value. A *p* value of < 0.05 was considered statistically significant.

## Additional files

Additional file 1: **Figure S1.** Screening ER-associated epigenetic markers with FLIM-FRET. (A) Representative raw FLIM images from the donor channel. (B) Corresponding normalized lifetime histogram of FLIM image in Fig. 1c from patient tissue array. Scale bar = 10 μM. (PDF 130 kb)

Additional file 2: **Figure S2.** Anacardic acid inhibits histone acetylation level. (A) MTT cell viability assay of MCF7 cells after 48 h treatment of anacardic acid at various concentrations, *n* > 5. (B) MTT cell viability assay of T47D cells after 48 h of treatment with 100 μM anacardic acid and 10 μM tamoxifen, *n* = 3. (C) Global H3k27ac quantification of combination treatment of 10 μM tamoxifen and 50 μM anacardic acid after 24 h treatment. (D) H4K12ac quantification from mice xenograft, TAM 4 mg kg^-1^, AA 1 mg kg^-1^, and TAM 4 mg kg^-1^ + AA 1 mg kg^-1^, *n* = 3. (E) Normalized H4K12ac quantification of both MCF7 cell and T47D cells after 24 h and 48 h of treatment with 100 μM anacardic acid, *n* = 3. (F) Western blot quantification of H4K12ac level. Histone protein from 100 μM AA treated MCF7 cell for 24 h. Data shown as mean ± s.d. *p < 0.05, **p < 0.01 compared with control group. (PDF 512 kb)

Additional file 3: **Figure S3.** Reduced FRET efficiency between ERα and histone acetylation marker after 80 μM anacardic acid treatment for 24 h. (A) Typical donor channel FLIM images of MCF7 cells treated with and without anacardic acid. Only fluorescence lifetime information was shown. Typical single cell lifetime histogram (B) without and (C) with anacardic acid treatment. Fluorescence lifetime histogram of ERα-ALEXA488 only (red); co-immunostaining of ERα-ALEXA488 and H4K12ac-ALEXA546 (blue) are shown parallel for comparison. A binning of 7 and 100 counts as threshold for background was applied for analysis. (D) TCSPC graph of typical single cell lifetime decay with ERα-H4K12ac interaction used for analysis. (E) FRET efficiency reduces non-specific level after anacardic acid exposure between ERα and H4K12ac/H3K27ac. *n*~20 cells. Scale bar = 5 μm. (PDF 433 kb)

Additional file 4: **Figure S4.** Non-specific HATi on screened histone acetylation marker shows minimal therapeutic effect. MTT cell viability assay after 48 h treatment of (A) MB3 and (B) CPTH2 at various concentrations. Date shown as mean ± s.d., *n* = 3. (C) H4K12ac quantification after 24 h treatment with 300 μM of either CPTH2 or MB3. *n* = 3, shown in mean ± s.d. (PDF 299 kb)

Additional file 5: **Figure S5.** Combination treatment based on FLIM-FRET screening. (A) MTT assays show treatment of 10 μM tamoxifen and 100 μM anacardic acid for 24. *n* = 3, *p < 0.05, **p < 0.01. (B) MTT assay shows treatment of 10 μM tamoxifen and 50 μM anacardic acid for 48 h from 2 independent assays (C) Combination treatment of TAM (4 mg kg^-1^) with AA (0.3 mg kg^-1^) did not show enhanced treatment effect in mice MCF7 cell xenograft. Mean ± s.e.m., *n* = 5. (D) qRT-PCR of TFF1, CCND1, and GREB1 genes from three different mice tumors. For each gene, left to right as control, TAM 4 mg kg^-1^, AA 1 mg kg^-1^, and TAM 4 mg kg^-1^ + AA 1 mg kg^-1^. *n* = 3 (PDF 294 kb)

Additional file 6: **Figure S6.** Co-presence of ERα and H4K12ac near ERE sites. qRT-PCR experiments were conducted with mice tumor for ERα and H4K12ac occupancy near TFF1/GREB1 ERE in CHIP samples. *n* = 2. mean ± s.d. (PDF 221 kb)

## Abbreviations

AA: Anacardic acid
ER: Estrogen receptor
ERE: Estrogen receptor element
FLIM-FRET: Fluorescence lifetime imaging-based Förster resonance energy transfer
H3K27ac: Histone 3 lysine27 acetylation
H4K12ac: Histone 4 lysine12 acetylation
HATi: Histone acetyltransferase inhibitor
MBD2: Methyl-CpG-binding domain protein 2
TAM: Tamoxifen

## References

[1] Al-Hajj M, Wicha MS, Benito-Hernandez A, Morrison SJ, Clarke MF (2003). Prospective identification of tumorigenic breast cancer cells. Proc Natl Acad Sci.

[2] DeSantis CE, Ma J, Goding Sauer A, Newman LA, Jemal A (2017). Breast cancer statistics, 2017, racial disparity in mortality by state. CA Cancer J Clin.

[3] DeSantis C, Ma J, Bryan L, Jemal A (2014). Breast cancer statistics, 2013. CA Cancer J Clin.

[4] Early Breast Cancer Trialists’ Collaborative G (2011). Relevance of breast cancer hormone receptors and other factors to the efficacy of adjuvant tamoxifen: patient-level meta-analysis of randomised trials. Lancet.

[5] Kohler BA, Sherman RL, Howlader N, Jemal A, Ryerson AB, Henry KA, Boscoe FP, Cronin KA, Lake A, Noone A-M (2015). Annual Report to the Nation on the Status of Cancer, 1975–2011, Featuring Incidence of Breast Cancer Subtypes by Race/Ethnicity, Poverty, and State. J Natl Cancer Inst.

[6] Amir E, Seruga B, Niraula S, Carlsson L, Ocaña A (2011). Toxicity of adjuvant endocrine therapy in postmenopausal breast cancer patients: a systematic review and meta-analysis. J Natl Cancer Inst.

[7] Vogel VG, Costantino JP, Wickerham D (2006). Effects of tamoxifen vs raloxifene on the risk of developing invasive breast cancer and other disease outcomes: the nsabp study of tamoxifen and raloxifene (star) p-2 trial. JAMA.

[8] Schlotter CM, Vogt U, Allgayer H, Brandt B (2008). Molecular targeted therapies for breast cancer treatment. Breast Cancer Res.

[9] Torrance CJ, Agrawal V, Vogelstein B, Kinzler KW (2001). Use of isogenic human cancer cells for high-throughput screening and drug discovery. Nat Biotechnol.

[10] Bleicher KH, Böhm H-J, Müller K, Alanine AI (2003). Hit and lead generation: beyond high-throughput screening. Nat Rev Drug Discov.

[11] Becker W (2012). Fluorescence lifetime imaging—techniques and applications. J Microsc.

[12] Miyagi T, Shiotani B, Miyoshi R, Yamamoto T, Oka T, Umezawa K, Ochiya T, Takano M, Tahara H (2014). DSE-FRET: a new anticancer drug screening assay for DNA binding proteins. Cancer Sci.

[13] Rogers MS, Cryan LM, Habeshian KA, Bazinet L, Caldwell TP, Ackroyd PC, Christensen KA (2012). A FRET-based high throughput screening assay to identify inhibitors of anthrax protective antigen binding to capillary morphogenesis gene 2 protein. PLoS One.

[14] Kumar S, Alibhai D, Margineanu A, Laine R, Kennedy G, McGinty J, Warren S, Kelly D, Alexandrov Y, Munro I (2011). FLIM FRET technology for drug discovery: automated multiwell-plate high-content analysis, multiplexed readouts and application in situ. Chemphyschem.

[15] Tian H, Ip L, Luo H, Chang DC, Luo KQ (2007). A high throughput drug screen based on fluorescence resonance energy transfer (FRET) for anticancer activity of compounds from herbal medicine. Br J Pharmacol.

[16] Mian OY, Wang SZ, Zhu SZ, Gnanapragasam MN, Graham L, Bear HD, Ginder GD (2011). Methyl binding domain protein 2 (MBD2) dependent proliferation and survival of breast cancer cells. Mol Cancer Res.

[17] Alvarado S, Wyglinski J, Suderman M, Andrews SA, Szyf M (2013). Methylated DNA binding domain protein 2 (MBD2) coordinately silences gene expression through activation of the microRNA hsa-mir-496 promoter in breast cancer cell line. PLoS One.

[18] Dekker FJ, van den Bosch T, Martin NI (2014). Small molecule inhibitors of histone acetyltransferases and deacetylases are potential drugs for inflammatory diseases. Drug Discov Today.

[19] Kutanzi K, Koturbash I, Kovalchuk O (2010). Reversibility of pre-malignant estrogen-induced epigenetic changes. Cell Cycle.

[20] Lupien M, Eeckhoute J, Meyer CA, Krum SA, Rhodes DR, Liu XS, Brown M (2009). Coactivator function defines the active estrogen receptor alpha cistrome. Mol Cell Biol.

[21] Sankari Nagarajan EB, Fischer A, Johnsen SA (2015). H4K12ac is regulated by estrogen receptor-alpha and is associated with BRD4 function and inducible transcription. Oncotarget.

[22] Nagarajan S, Hossan T, Alawi M, Najafova Z, Indenbirken D, Bedi U, Taipaleenmäki H, Ben-Batalla I, Scheller M, Loges S (2014). Bromodomain protein BRD4 is required for estrogen receptor-dependent enhancer activation and gene transcription. Cell Rep.

[23] Jangal M, Couture J-P, Bianco S, Magnani L, Mohammed H, Gévry N (2014). The transcriptional co-repressor TLE3 suppresses basal signaling on a subset of estrogen receptor α target genes. Nucleic Acids Res.

[24] Murakami S, Nagari A, Kraus WL (2017). Dynamic assembly and activation of estrogen receptor alpha enhancers through coregulator switching. Genes Dev.

[25] Suzuki J, Chen YY, Scott GK, Devries S, Chin K, Benz CC, Waldman FM, Hwang ES (2009). Protein acetylation and histone deacetylase expression associated with malignant breast cancer progression. Clin Cancer Res.

[26] Wong CC, Qian Y, Yu J (2017). Interplay between epigenetics and metabolism in oncogenesis: mechanisms and therapeutic approaches. Oncogene.

[27] Karsli-Ceppioglu S, Dagdemir A, Judes G, Lebert A, Penault-Llorca F, Bignon YJ, Bernard-Gallon D (2017). The epigenetic landscape of promoter genome-wide analysis in breast cancer. Sci Rep.

[28] Fisher B, Costantino JP, Wickerham DL, Redmond CK, Kavanah M, Cronin WM, Vogel V, Robidoux A, Dimitrov N, Atkins J (1998). Tamoxifen for prevention of breast cancer: report of the National Surgical Adjuvant Breast and Bowel Project P-1 study. J Natl Cancer Inst.

[29] Schultz DJ, Wickramasinghe NS, Ivanova MM, Isaacs SM, Dougherty SM, Imbert-Fernandez Y, Cunningham AR, Chen C, Klinge CM (2010). Anacardic acid inhibits estrogen receptor α–DNA binding and reduces target gene transcription and breast cancer cell proliferation. Mol Cancer Ther.

[30] Schild-Hay LJ, Leil TA, Divi RL, Olivero OA, Weston A, Poirier MC (2009). Tamoxifen induces expression of immune response-related genes in cultured normal human mammary epithelial cells. Cancer Res.

[31] Dietze EC, Caldwell LE, Grupin SL, Mancini M, Seewaldt VL (2001). Tamoxifen but not 4-hydroxytamoxifen initiates apoptosis in p53(−) normal human mammary epithelial cells by inducing mitochondrial depolarization. J Biol Chem.

[32] Carvalho AL, Annoni R, Silva PR, Borelli P, Fock RA, Trevisan MT, Mauad T (2011). Acute, subacute toxicity and mutagenic effects of anacardic acids from cashew (Anacardium occidentale Linn.) in mice. J Ethnopharmacol.

[33] Sung B, Pandey MK, Ahn KS, Yi T, Chaturvedi MM, Liu M, Aggarwal BB (2008). Anacardic acid (6-nonadecyl salicylic acid), an inhibitor of histone acetyltransferase, suppresses expression of nuclear factor-kappaB-regulated gene products involved in cell survival, proliferation, invasion, and inflammation through inhibition of the inhibitory subunit of nuclear factor-kappaB alpha kinase, leading to potentiation of apoptosis. Blood.

[34] Chen Y, Damayanti NP, Irudayaraj J, Dunn K, Zhou FC (2014). Diversity of two forms of DNA methylation in the brain. Front Genet.

[35] Cui Y, Irudayaraj J (2015). Dissecting the behavior and function of MBD3 in DNA methylation homeostasis by single-molecule spectroscopy and microscopy. Nucleic Acids Res.

[36] Mendonca A, Chang EH, Liu W, Yuan C (2014). Hydroxymethylation of DNA influences nucleosomal conformation and stability in vitro. Biochim Biophys Acta.

[37] Chen J, Miller A, Kirchmaier AL, Irudayaraj JMK (2012). Single-molecule tools elucidate H2A.Z nucleosome composition. J Cell Sci.

[38] Chen J, Irudayaraj J (2010). Fluorescence lifetime cross correlation spectroscopy resolves EGFR and antagonist interaction in live cells. Anal Chem.

[39] Jeong KW, Kim K, Situ AJ, Ulmer TS, An W, Stallcup MR (2011). Recognition of enhancer element-specific histone methylation by TIP60 in transcriptional activation. Nat Struct Mol Biol.

[40] Townson SM, Kang K, Lee AV, Oesterreich S (2006). Novel role of the RET finger protein in estrogen receptor-mediated transcription in MCF-7 cells. Biochem Biophys Res Commun.

