# Supplementary material for: Epigenetic biomarker screening by FLIM-FRET for combination therapy in ER+ breast cancer
Source: Clin Epigenetics. 2019 Jan 30;11:16. doi: 10.1186/s13148-019-0620-6 (PMC6354376; doi:10.1186/s13148-019-0620-6)
Supplement: Supplementary file 1 — Figure S1. Screening ER-associated epigenetic markers with FLIM-FRET. (A) Representative raw FLIM images from the donor channel. (B) Corresponding normalized lifetime histogram of FLIM image in Fig. 1c from patient tissue array. Scale bar = 10 μM. (PDF 130 kb) [file 13148_2019_620_MOESM1_ESM.pdf]

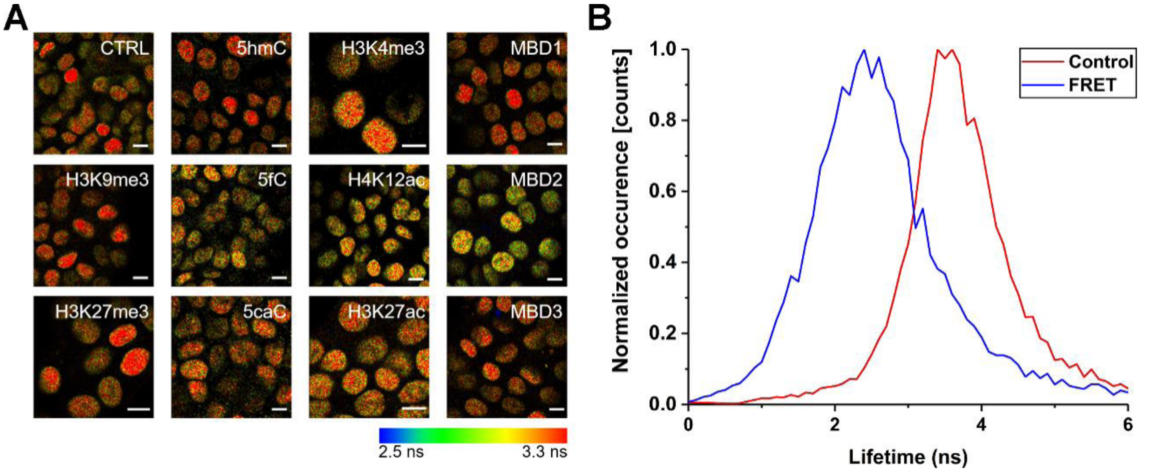

Figure S1. Screening ER-associated epigenetic markers with FLIM-FRET. (A) Representative raw FLIM images from the donor channel. (B) Corresponding normalized lifetime histogram of FLIM image in Figure 1(C) from patient tissue array. Scale bar = 10 M.
