# Supplementary material for: Epigenetic biomarker screening by FLIM-FRET for combination therapy in ER+ breast cancer
Source: Clin Epigenetics. 2019 Jan 30;11:16. doi: 10.1186/s13148-019-0620-6 (PMC6354376; doi:10.1186/s13148-019-0620-6)
Supplement: Supplementary file 2 — Figure S2. Anacardic acid inhibits histone acetylation level. (A) MTT cell viability assay of MCF7 cells after 48 h treatment of anacardic acid at various concentrations, n > 5. (B) MTT cell viability assay of T47D cells after 48 h of treatment with 100 μM anacardic acid and 10 μM tamoxifen, n = 3. (C) Global H3k27ac quantification of combination treatment of 10 μM tamoxifen and 50 μM anacardic acid after 24 h treatment. (D) H4K12ac quantification from mice xenograft, TAM 4 mg kg-1, AA 1 mg kg-1, and TAM 4 mg kg-1 + AA 1 mg kg-1, n = 3. (E) Normalized H4K12ac quantification of both MCF7 cell and T47D cells after 24 h and 48 h of treatment with 100 μM anacardic acid, n = 3. (F) Western blot quantification of H4K12ac level. Histone protein from 100 μM AA treated MCF7 cell for 24 h. Data shown as mean ± s.d. *p < 0.05, **p < 0.01 compared with control group. (PDF 512 kb) [file 13148_2019_620_MOESM2_ESM.pdf]

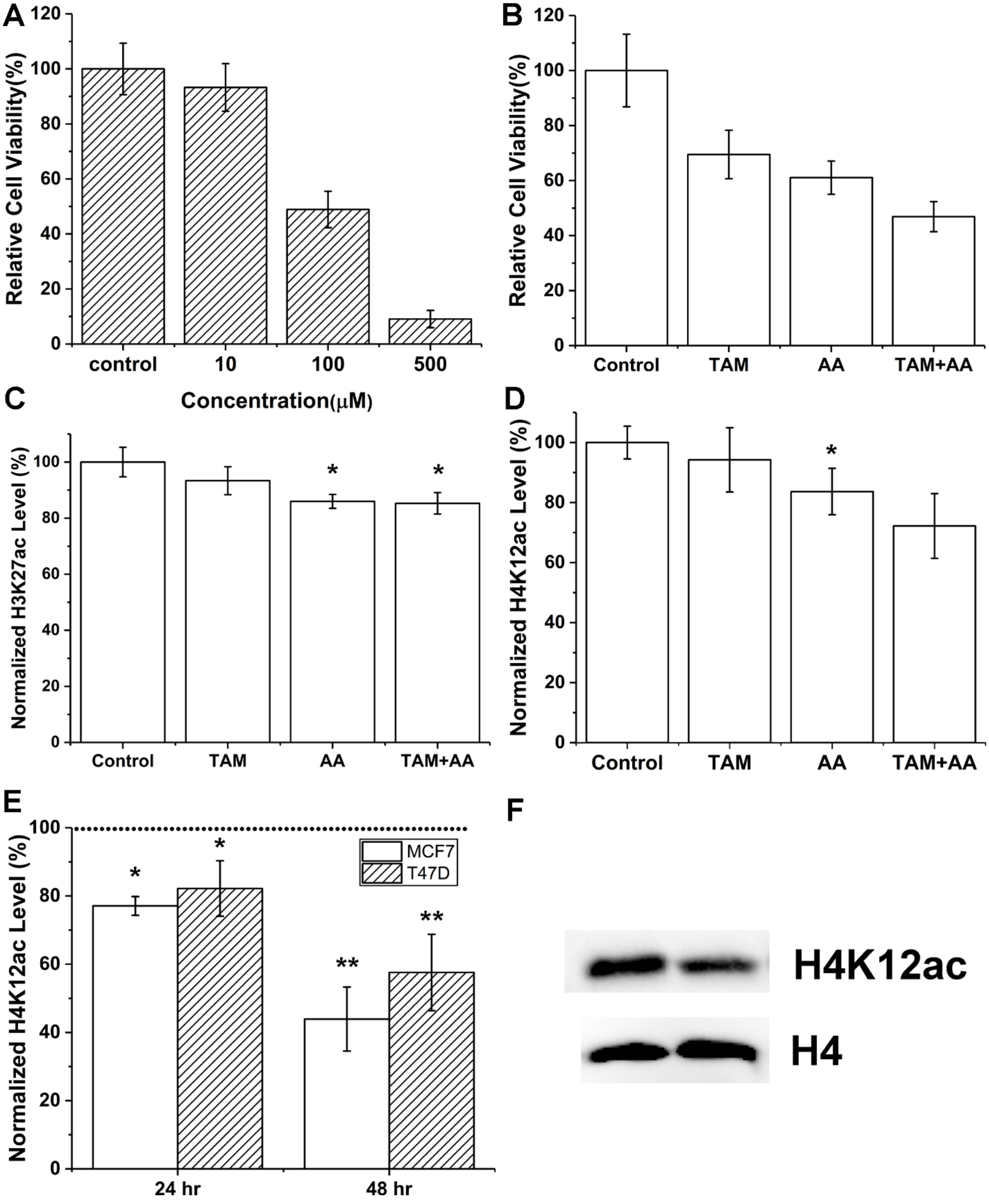

Figure S2. Anacardic acid inhibits histone acetylation level. (A) MTT cell viability assay of MCF7 cells after 48 h treatment of anacardic acid at various concentrations, n>5. (B) MTT cell viability assay of T47D cells after 48 h of treatment with 100 μM anacardic acid and 10 μM tamoxifen, n=3. (C) Global H3k27ac quantification of combination treatment of 10 μM tamoxifen and 50 μM anacardic acid after 24 hr

treatment. (D) H4K12ac quantification from mice xenograft, TAM 4mg kg^{-1}, AA 1mg kg^{-1} and TAM 4mg kg^{-1}+AA 1mg kg^{-1}, n=3. (E) Normalized H4K12ac quantification of both MCF7 cell and T47D cells after 24h and 48 hr of treatment with 100 μM anacardic acid, n=3. (F) Western blot quantification of H4K12ac level. Histone protein from 100 μM AA treated MCF7 cell for 24hr. Data shown as mean ± s.d. *: $p<0.05$, **: $p<0.01$ compared with control group.
