# Supplementary material for: Epigenetic biomarker screening by FLIM-FRET for combination therapy in ER+ breast cancer
Source: Clin Epigenetics. 2019 Jan 30;11:16. doi: 10.1186/s13148-019-0620-6 (PMC6354376; doi:10.1186/s13148-019-0620-6)
Supplement: Supplementary file 3 — Figure S3. Reduced FRET efficiency between ERα and histone acetylation marker after 80 μM anacardic acid treatment for 24 h. (A) Typical donor channel FLIM images of MCF7 cells treated with and without anacardic acid. Only fluorescence lifetime information was shown. Typical single cell lifetime histogram (B) without and (C) with anacardic acid treatment. Fluorescence lifetime histogram of ERα-ALEXA488 only (red); co-immunostaining of ERα-ALEXA488 and H4K12ac-ALEXA546 (blue) are shown parallel for comparison. A binning of 7 and 100 counts as threshold for background was applied for analysis. (D) TCSPC graph of typical single cell lifetime decay with ERα-H4K12ac interaction used for analysis. (E) FRET efficiency reduces non-specific level after anacardic acid exposure between ERα and H4K12ac/H3K27ac. n~20 cells. Scale bar = 5 μm. (PDF 433 kb) [file 13148_2019_620_MOESM3_ESM.pdf]

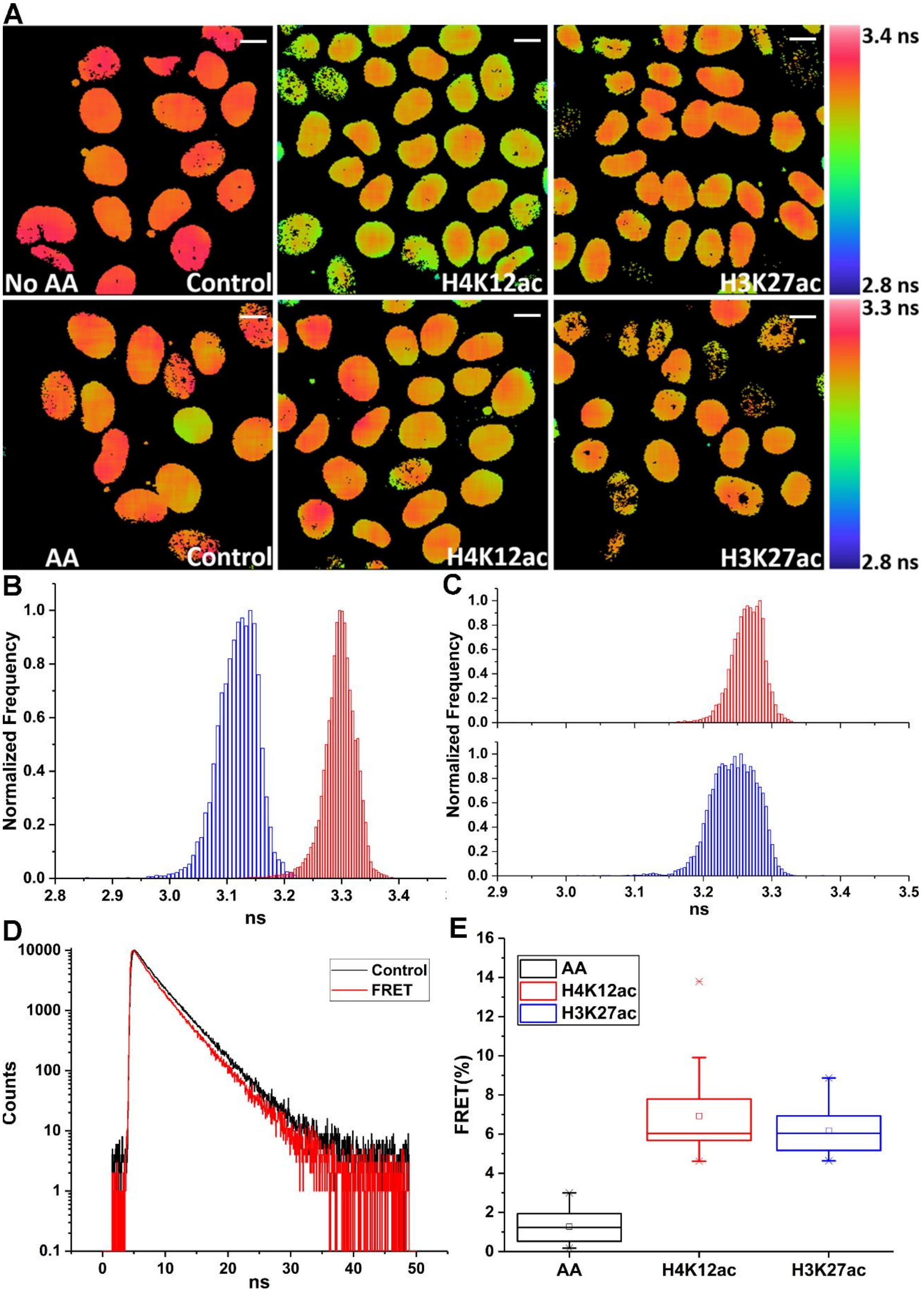

Figure S3. Reduced FRET efficiency between ERα and histone acetylation marker after 80 μM anacardic acid treatment for 24h. (A) Typical donor channel FLIM images

of MCF7 cells treated with and without anacardic acid. Only fluorescence lifetime information was shown. Typical single cell lifetime histogram (B) without and (C) with anacardic acid treatment. Fluorescence lifetime histogram of ERα-ALEXA488 only (red); co-immunostaining of ERα-ALEXA488 and H4K12ac-ALEXA546 (blue) are shown parallel for comparison. A binning of 7, and 100 counts as threshold for background was applied for analysis. (D) TCSPC graph of typical single cell lifetime decay with ERα-H4K12ac interaction used for analysis. (E) FRET efficiency reduces non-specific level after anacardic acid exposure between ERα and H4K12ac/H3K27ac. n~20 cells. Scale bar=5 μm.
