# Supplementary material for: Epigenetic biomarker screening by FLIM-FRET for combination therapy in ER+ breast cancer
Source: Clin Epigenetics. 2019 Jan 30;11:16. doi: 10.1186/s13148-019-0620-6 (PMC6354376; doi:10.1186/s13148-019-0620-6)
Supplement: Supplementary file 4 — Figure S4. Non-specific HATi on screened histone acetylation marker shows minimal therapeutic effect. MTT cell viability assay after 48 h treatment of (A) MB3 and (B) CPTH2 at various concentrations. Date shown as mean ± s.d., n = 3. (C) H4K12ac quantification after 24 h treatment with 300 μM of either CPTH2 or MB3. n = 3, shown in mean ± s.d. (PDF 299 kb) [file 13148_2019_620_MOESM4_ESM.pdf]

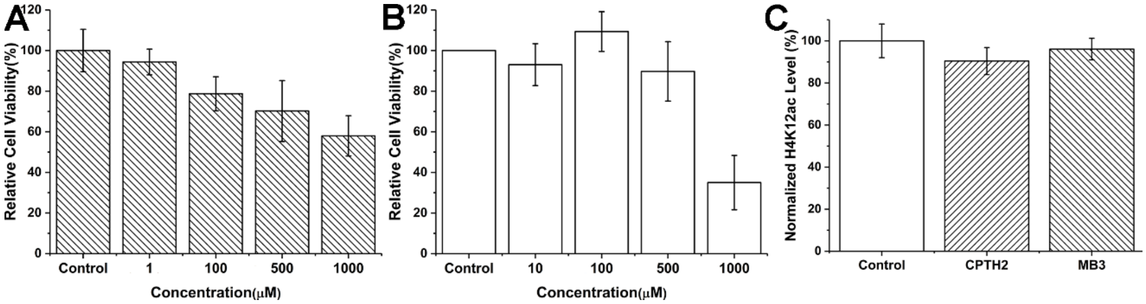

Figure S4. Non-specific HATi on screened histone acetylation marker shows minimal therapeutic effect. MTT cell viability assay after 48 h treatment of (A) MB3 and (B) CPTH2 at various concentrations. Date shown as mean ± s.d., n=3. (C) H4K12ac quantification after 24 h treatment with 300 μM of either CPTH2 or MB3. n=3, shown in mean ± s.d.
