# Supplementary material for: Epigenetic biomarker screening by FLIM-FRET for combination therapy in ER+ breast cancer
Source: Clin Epigenetics. 2019 Jan 30;11:16. doi: 10.1186/s13148-019-0620-6 (PMC6354376; doi:10.1186/s13148-019-0620-6)
Supplement: Supplementary file 5 — Figure S5. Combination treatment based on FLIM-FRET screening. (A) MTT assays show treatment of 10 μM tamoxifen and 100 μM anacardic acid for 24. n = 3, *p < 0.05, **p < 0.01. (B) MTT assay shows treatment of 10 μM tamoxifen and 50 μM anacardic acid for 48 h from 2 independent assays (C) Combination treatment of TAM (4 mg kg-1) with AA (0.3 mg kg-1) did not show enhanced treatment effect in mice MCF7 cell xenograft. Mean ± s.e.m., n = 5. (D) qRT-PCR of TFF1, CCND1, and GREB1 genes from three different mice tumors. For each gene, left to right as control, TAM 4 mg kg-1, AA 1 mg kg-1, and TAM 4 mg kg-1 + AA 1 mg kg-1. n = 3 (PDF 294 kb) [file 13148_2019_620_MOESM5_ESM.pdf]

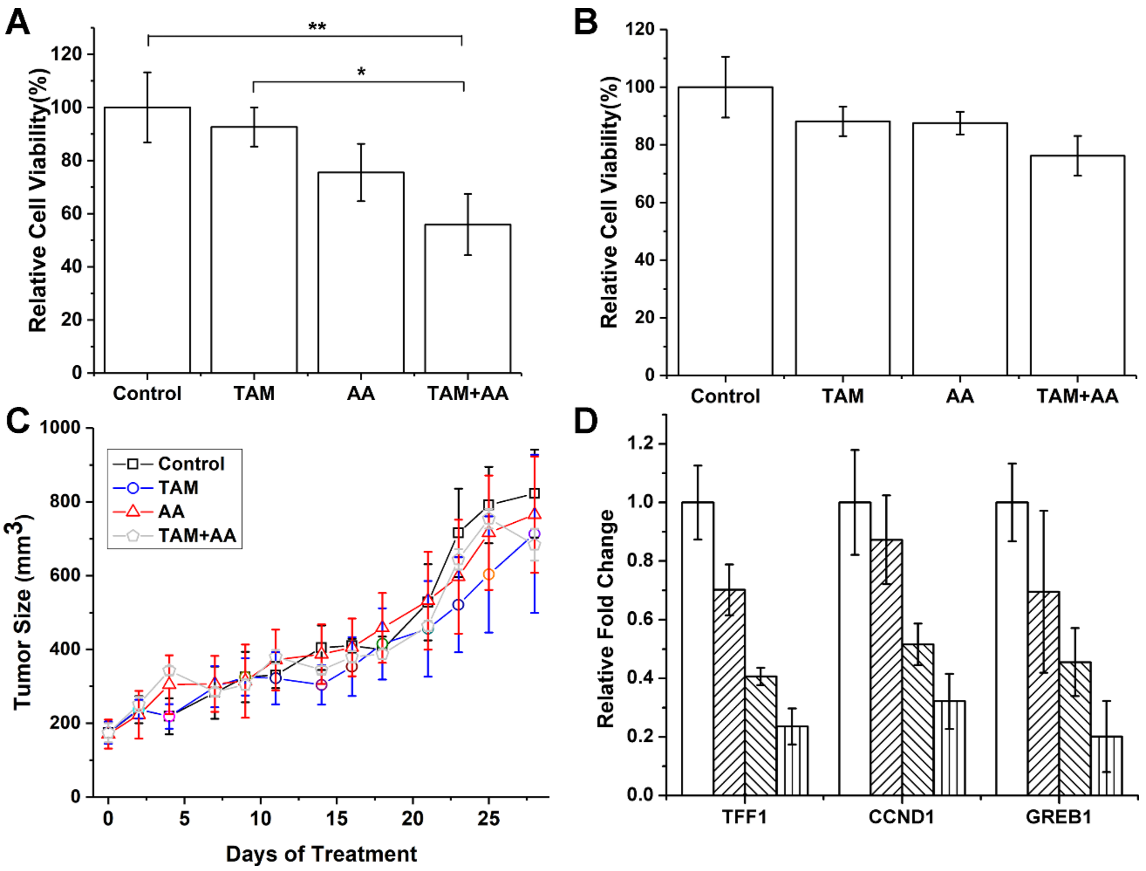


**Figure S5. Combination treatment based on FLIM-FRET screening.** (A) MTT assays shows treatment of 10 μM tamoxifen and 100 μM anacardic acid for 24 . n=3, * $p<0.05$, ** $p<0.01$. (B) MTT assay shows treatment of 10 μM tamoxifen and 50 μM anacardic acid for 48 h from 2 independent assays (C) Combination treatment of TAM (4mg $kg^{-1}$) with AA (0.3mg $kg^{-1}$) did not show enhanced treatment effect in mice MCF7 cell xenograft. Mean ± s.e.m, n=5. (D) qRT-PCR of TFF1, CCND1 and GREB1 genes from three different mice tumors. For each gene, left to right as Control, TAM 4mg $kg^{-1}$, AA 1mg $kg^{-1}$ and TAM 4mg $kg^{-1}$+AA 1mg $kg^{-1}$. n=3
