# Supplementary material for: Epigenetic biomarker screening by FLIM-FRET for combination therapy in ER+ breast cancer
Source: Clin Epigenetics. 2019 Jan 30;11:16. doi: 10.1186/s13148-019-0620-6 (PMC6354376; doi:10.1186/s13148-019-0620-6)
Supplement: Supplementary file 6 — Figure S6. Co-presence of ERα and H4K12ac near ERE sites. qRT-PCR experiments were conducted with mice tumor for ERα and H4K12ac occupancy near TFF1/GREB1 ERE in CHIP samples. n = 2. mean ± s.d. (PDF 221 kb) [file 13148_2019_620_MOESM6_ESM.pdf]

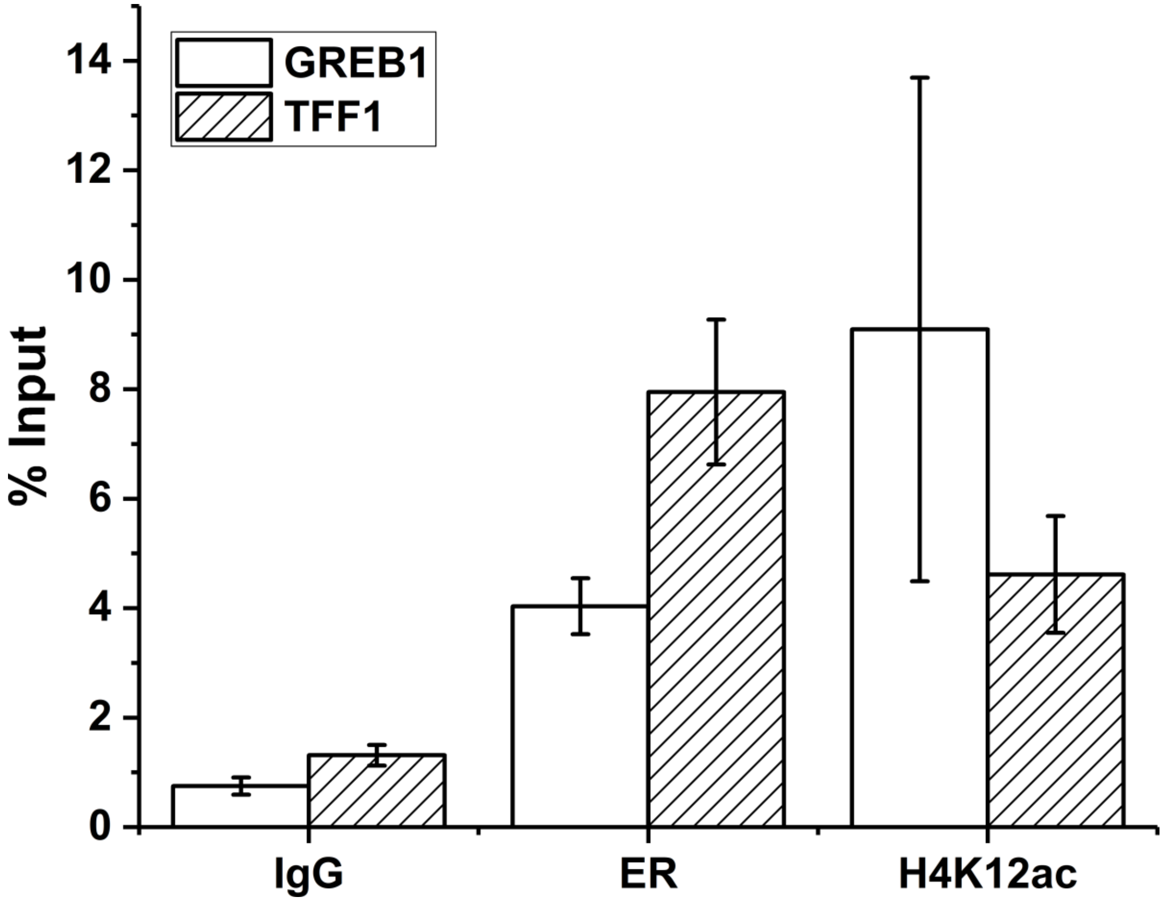

Figure S6. Co-prescence of ERα and H4K12ac near ERE sites. qRT-PCR experiments were conducted with mice tumor for ERα and H4K12ac occupancy near TFF1/GREB1 ERE in CHIP samples. n=2. mean ± s.d.
